# Mesenchymal Stem Cell-Derived Extracellular Vesicles: Roles in Tumor Growth, Progression, and Drug Resistance

**DOI:** 10.1155/2017/1758139

**Published:** 2017-03-09

**Authors:** Xiaoyan Zhang, Huaijun Tu, Yazhi Yang, Lijun Fang, Qiong Wu, Jian Li

**Affiliations:** ^1^The Key Laboratory of Hematology of Jiangxi Province, The Department of Hematology, The Second Affiliated Hospital of Nanchang University, 1 Minde Road, Nanchang, Jiangxi 330006, China; ^2^Basic Medical School, Nanchang University, 465 Bayi Road, Nanchang, Jiangxi 330006, China; ^3^Graduate School of Medicine, Nanchang University, 465 Bayi Road, Nanchang, Jiangxi 330006, China; ^4^The Department of Neurology, The Second Affiliated Hospital of Nanchang University, 1 Minde Road, Nanchang, Jiangxi 330006, China

## Abstract

Mesenchymal stem cells (MSCs) are ubiquitously present in many tissues. Due to their unique advantages, MSCs have been widely employed in clinical studies. Emerging evidences indicate that MSCs can also migrate to the tumor surrounding stroma and exert complex effects on tumor growth and progression. However, the effect of MSCs on tumor growth is still a matter of debate. Several studies have shown that MSCs could favor tumor growth. On the contrary, other groups have demonstrated that MSCs suppressed tumor progression. Extracellular vesicles have emerged as a new mechanism of cell-to-cell communication in the development of tumor diseases. MSCs-derived extracellular vesicles (MSC-EVs) could mimic the effects of the mesenchymal stem cells from which they originate. Different studies have reported that MSC-EVs may exert various effects on the growth, metastasis, and drug response of different tumor cells by transferring proteins, messenger RNA, and microRNA to recipient cells. In the present review, we summarize the components of MSC-EVs and discuss the roles of MSC-EVs in different malignant diseases, including the related mechanisms that may account for their therapeutic potential. MSC-EVs open up a promising opportunity in the treatment of cancer with increased efficacy.

## 1. Introduction

Mesenchymal stem cells (MSCs) are multipotent cells that can differentiate into various cell types of the mesodermal germ layer. MSCs can also be recruited to the sites of inflammation and tissue repair [1–5]. In addition, they possess multiple biological functions including multilineage differentiation, immunosuppression, and tissue-repair promotion [6–8]. Due to these unique advantages, MSCs have been widely employed in clinical studies [9–15], such as spinal cord injuries, cardiovascular diseases, type I diabetes mellitus, hepatic cirrhosis, and Alzheimer's disease (https://clinicaltrials.gov/).

Recent studies have demonstrated that MSCs can also migrate to the tumor stroma, contributing to the formation of the tumor microenvironment [16–20]. Several studies have shown that MSCs could favor tumor growth directly by producing growth factors or promoting tumor vascularization [21–24]. On the contrary, other groups demonstrated that MSCs suppressed tumor progression [25–29]. However, the exact mechanisms of these opposite effects remain unclear [30]. A large body of MSCs research has focused on MSC-derived extracellular vesicles (MSC-EVs) and shown that MSC-EVs have functions similar to those of MSCs [31–38], such as repairing tissue damage, suppressing inflammatory responses, and promoting angiogenesis.

MSC-EVs could also be involved in the effects of MSCs on tumor growth and behavior. Several studies describing the influence of MSC-EVs on tumor growth have been reported. Thus, it is reasonable to postulate that MSC-EVs transport key MSC-associated molecules which change the physiology of target cells in a specific manner. MSC-EVs have emerged as a new mechanism of cell-to-cell communication in the development and growth of human malignancies.

In this article, first we will review the composition of MSC-EVs which will be classified based on their molecular contents into four groups: proteins, messenger RNAs (mRNAs), microRNAs (miRNAs), and others. Then the effects of MSC-EVs on cancer development and progression will be highlighted. Finally, we will address the possible molecular mechanisms underlying MSC-EVs-mediated therapeutic effects.

## 2. Characterization of MSC-EVs

MSC-EVs are a heterogeneous population that mainly include exosomes, microvesicle particles (also known as ectosomes), and apoptotic bodies. Exosomes have a diameter of 30–100 nm, secreted upon fusion of multivesicular endosomes with the plasma membranes. Microvesicle particles are usually larger than exosomes (100–1000 nm), resulting from outward budding of plasma membrane. These vesicles are shed into the extracellular space constitutively, or as consequence to physical or chemical stress, hypoxia, and soluble agonists [61, 62]. MSC-EVs contain membranes and cytoplasmic constituents of the original cells. MSC-EVs membranes are enriched in sphingomyelin, cholesterol, and ceramide [63]. They are positive for surface markers of MSCs (CD13, CD90, CD29, CD44, CD73, and CD105), but negative for the hematopoietic system-related markers (CD34 and CD45). Moreover, MSC-EVs also express the two characteristic markers of EVs, CD81 and CD63 [39, 40]. According to the different origins of MSCs, MSC-EVs have been divided into different subtypes: human bone marrow-derived MSC-EVs (hBMSC-EVs), human adipose-derived MSC-EVs (hAMSC-EVs), human umbilical cord MSC-EVs (hUCMSC-EVs), mouse bone marrow-derived MSC-EVs (mBMSC-EVs), porcine adipose tissue-derived MSC-EVs (pAMSC-EVs), and so forth. It is difficult to distinguish different subpopulations of MSC-EVs due to their overlapping size, density, and composition [64].

## 3. Cargoes of MSC-EVs

**Figure 1:**
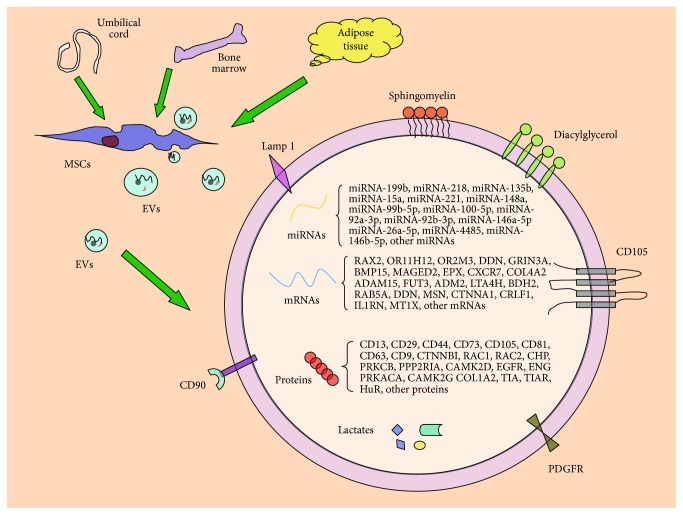
Composition of MSC-EVs. MSC-EVs carry a variety of molecules including proteins, mRNAs, miRNAs, and lipids. Transfer of these biological materials into adjacent or distant cells may influence the behavior of the recipient cells.

Several studies have revealed that MSC-EVs contain proteins, lipids, and genetic materials, such as mRNAs and miRNAs [65] (Figure 1). Transfer of these biological materials into adjacent or distant cells may influence the behavior of the recipient cells [32, 36, 66].

### 3.1. Protein Contents of MSC-EVs

Researchers have identified 730 proteins in hBMSC-EVs according to liquid chromatography-tandem mass spectrometry analysis [39]. Functional analysis of the hBMSC-EVs proteome indicates that these proteins are involved in cell proliferation, adhesion, migration, and self-renewal, mainly including surface receptors, signaling molecules, cell adhesion molecules, and MSCs-associated antigens (CD9, CD63, CD81, CD109, CD151, CD248, and CD276) (Table 1). Among these molecules, CD63, CD9, and CD81 are the specific exosomal markers [41]. Moreover, MSC-EVs express some surface molecules, such as CD29, CD73, CD44, and CD105, but do not express the hematopoietic system-related markers, CD34 and CD45 [40]. Tumor supportive factors such as PDGFR-*β*, TIMP-1, and TIMP-2 were also identified in BMSC-EVs [41]. In addition, hAMSC-EVs carried enzymatically active Neprilysin [42], which degrade intracellular and extracellular *β*-amyloid peptide in neuroblastoma cell lines.

Another study showed that MSC-EVs contained ribonucleoproteins, such as T cell internal antigen-1 (TIA), TIA-1-related (TIAR) and AU-rich element binding protein (Hu R), argonaute2 (Ago2), staufen1 (Stau1) and staufen2 (Stau2) proteins, which are implicated in the transport and stability of mRNA [43]. Researchers also discovered that Wnt4 [44], angiogenin, basic fibroblast growth factors (bFGF), vascular endothelial growth factor (VEGF), monocyte chemotactic protein-1 (MCP-1), the receptor-2 for vascular endothelial growth factor (VEGF R2), insulin like growth factor I (IGF-I), Tie-2/TEK, and interleukin-6 (IL-6) [45] were highly expressed in hUCMSC-EVs, which could promote *β*-catenin nuclear translocation and enhance angiogenesis. It was also reported that MSC exosomes had all seven *α*- and seven *β*-chains of the 20S proteasome. The 20S proteasome was thought to reduce accumulation of denatured or misfolded proteins [67].

### 3.2. mRNA

Besides proteins, one of the most distinct features of MSC-EVs is that they also contain nucleic acids, including mRNAs and miRNAs [65]. mRNAs and miRNAs can be transferred into a recipient cell located in the tumor microenvironment or at distant sites via fusion of MSC-EVs with the target cell membrane.

It was demonstrated that the mRNAs present in EVs are associated with the mesenchymal phenotype and with several cell functions related to the control of cell differentiation (RAX2, OR11H12, OR2M3, DDN, and GRIN3A), transcription (CLOCK, IRF6, RAX2, TCFP2, and BCL6B), proliferation (SENP2, RBL1, CDC14B, and S100A13), cytoskeleton (DDN, MSN, and CTNNA1), metabolism (ADAM15, FUT3, ADM2, LTA4H, BDH2, and RAB5A) [47], and cell immune regulation (CRLF1, IL1RN, and MT1X) (Table 2). Furthermore, in an in vitro model of renal toxic injury, MSC-EVs were shown to contain mRNA for the insulin growth factor 1 (IGF-1) receptor. Transfer of IGF-1 receptor mRNA through MSC-EVs induced proliferation of proximal tubular cells [46].

In EVs from porcine adipose tissue-derived MSCs, researchers found distinct classes of RNAs were selectively expressed using high-throughput RNA sequencing [48]. EVs preferentially express mRNAs for angiogenesis, adipogenesis, Golgi apparatus, and transcription factors associated with alternative splicing, apoptosis, and chromosome organization. EVs also express genes involved in TGF-*β* signaling (TGFB1, TGFB3, FURIN, and ENG).

### 3.3. MicroRNA

In addition to mRNAs, MSC-EVs have been shown to contain miRNAs as well (Table 3). miRNAs are small noncoding RNAs containing 22 nucleotides [68]. After internalization by target cells, these miRNAs may function as either tumor suppressors or oncogenes, targeting specific mRNAs to mediate inhibition of translation [69].

It has been shown that 79 mature miRNAs could be detected in BMSC-EVs using miRNA arrays [49]. Among these miRNAs, five (miRNA-199b, miRNA-218, miRNA-148a, miRNA-135b, and miRNA-221) were differentially expressed at different time points in BMSC-EVs during osteogenic differentiation. Researchers have also analyzed the miRNA profile of EVs released by two different sources: AMSCs and BMSCs. The study has revealed that MSC-EVs mainly contain mature transcripts. The most expressed miRNAs in AMSC-EVs and BMSC-EVs are highly similar, but their relative proportions are different, raising the possibility that AMSC-EVs and BMSC-EVs may transfer different information [53, 70]. In contrast, EVs secreted by human embryonic stem cell-derived MSCs (hEMSCs-EVs) were enriched in precursor miRNAs rather than mature miRNAs [71]. This suggested that the EVs released by different MSCs might preferentially enclose different forms of miRNA.

Likewise, some other miRNAs, such as miRNA-15a [50], miRNA-16 [52], miRNA-21, miRNA-34a, and miRNA-191 [41, 72], have been identified in MSC-EVs and shown to prevent apoptosis, promote cellular growth [73], reduce cardiac fibrosis [74], and inhibit tumor growth [75] by regulating their target genes in recipient cells. While these miRNAs are not randomly sorted into the MSC-EVs, some miRNAs are present only in the original cells, but not in the MSC-EVs. However, some certain miRNAs are selectively sorted into the MSC-EVs, which are undetectable in the original MSCs, such as miRNA-564, miRNA-223, and miRNA-451. The specific mechanism of MSC-EVs content sorting is not clear.

### 3.4. Lipid and Other Contents of MSC-EVs

Our knowledge on the lipid composition of MSC-EVs is quite limited. Only a few studies confirmed high level of bioactive lipids such as diacylglycerol and sphingomyelin but trace amounts of dihydroceramide and *α*-hydroxy-ceramide in MSC-EVs. Furthermore, small molecule metabolite assays have demonstrated the presence of lactic acid and glutamic acid in EVs [41].

## 4. MSC-EVs Inhibit Proliferation and Promote the Apoptosis of Tumor Cells

The role of MSC-EVs in tumor proliferation has been well documented. However, the mechanisms by which MSC-EVs inhibit tumor growth are still uncertain. It has been demonstrated that MSC-EVs inhibited the proliferation of HepG2 hepatoma, Kaposi's sarcoma (KS), and Skov-3 ovarian cancer cell lines by blocking cell cycle progression in the G0/G1 phase [54]. Gene array profiles showed that the genes related to antiproliferative pathway were upregulated, such as GTP-binding RAS-like 3 (DIRAS3), retinoblastoma-like 1 (Rbl-1), and cyclin-dependent kinase inhibitor 2B transcript (CDKN2B), but different genes were modulated in various cancer cell lines. Moreover, EVs could induce apoptosis in HepG2 and Kaposi cells, as demonstrated by TUNEL assay. In contrast, EVs induced necrosis not apoptosis in Skov-3 cells and in vivo intratumor administration of EVs in established tumors generated by subcutaneous injection of these cell lines in SCID mice significantly inhibited tumor growth.

A similar effect was observed in EVs derived from human cord blood Wharton's jelly MSCs (hWJMSC-EVs) [55]. hWJMSC-EVs abolished T24 bladder tumor proliferation via G0/G1 phase arrest in a dose-dependent manner and induced apoptosis in T24 cells in vitro and in vivo. The antiproliferative and proapoptotic effects were mainly mediated by restraining phosphorylation of Akt, upregulation of p-p53, and activation of caspase cascade (caspase-3 cleavage).

Another recent paper described the effect of murine MSC-EVs on the expression of VEGF in mouse breast cancer cell line (4T1). It demonstrated that murine MSC-EVs significantly downregulated the expression of VEGF in a dose-dependent manner, causing inhibition of angiogenesis in vitro and in vivo. Additionally, miRNA-16 shuttled by MSC-EVs was partially responsible for the antiangiogenic effect of MSC-EVs [52].

In addition, it was reported that in hematological malignancies normal BMSC-EVs inhibited the growth of multiple myeloma (MM) cells, while MM BMSC-EVs promoted MM tumor growth [50]. Further study found that normal and MM BMSC-EVs differed in their protein and miRNA contents, with higher expression of cytokines, oncogenic proteins, and protein kinases in MM BMSC-EVs, but lower level of miRNA-15a. On the basis of this information, MSC-EVs could therefore exert either antiproliferation or proapoptotic effects on tumor cells (Table 4).

## 5. MSC-EVs Promote the Growth and Metastasis of Tumor Cells

The tumor growth promoting effects of MSC-EVs have also been suggested by various reports. For instance, researchers have found that MSC-EVs could increase tumor growth in BALB/c nu/nu mice xenograft model by enhancing VEGF expression through activation of extracellular signal regulated kinase 1/2 (ERK1/2) and p38 MAPK pathway [56]. Inhibition of ERK1/2 activation could reverse the increase of VEGF level by MSC-EVs. However, the proproliferative effect on cancer cells was not observed in vitro, and there were no differences in the percentage of cells in the G0/G1, S, and G2/M phases between EV-treated and untreated cells. These findings suggest that MSC-EVs do not directly stimulate proliferation of cancer cells in vitro but instead induce activation of an angiogenesis program that could favor tumor engraftment and growth.

MSC-EVs can also promote the metastasis of the breast cancer cell line MCF7 by activating the Wnt pathway. In a study on MM, researchers found that BMSC-EVs could promote proliferation, survival, and metastasis of myeloma cells. p38, p53, c-Jun N-terminal kinase, and Akt pathways in MM cells were influenced by BMSC-EVs [57].

In addition, Du et al. have reported that hWJMSC-EVs promoted the growth and migration of human renal cell carcinoma (RCC) cells both in vitro and in vivo. EVs facilitated the progression of cell cycle from G0/G1 to S. The mechanisms underlying this effect were suggested to be transfer of RNA material by EVs to induce hepatocyte growth factor (HGF) expression in RCC and activate Akt and ERK1/2 signaling pathways. Use of c-Met inhibitors can abrogate the activation of AKT and ERK1/2 signaling in 786-0 cells [58]. Interestingly, the same group has demonstrated the antiproliferative and proapoptotic effects of hWJMSC-EVs on bladder cancer cells [74].

**Figure 2:**
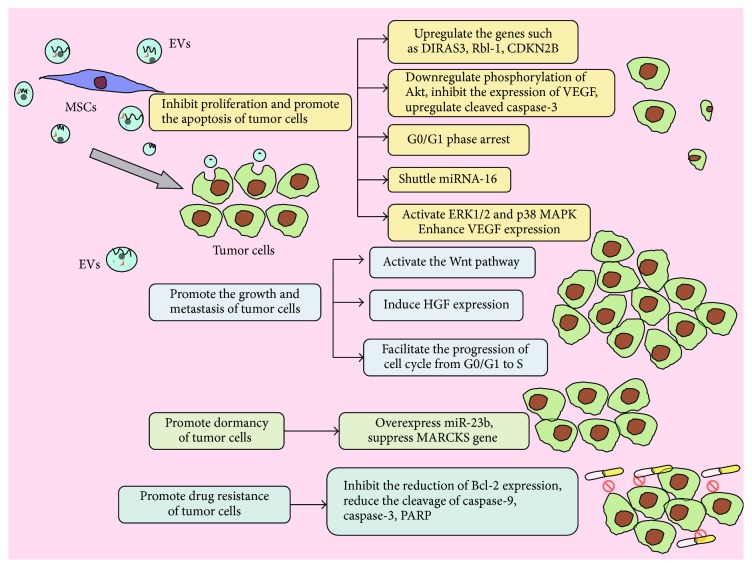
The different effects of MSC-EVs on the growth, metastasis, and drug response of different tumor cells.

Taken above findings together, the same EVs can have opposite effects on different tumors (Figure 2). The specific mechanism is not precisely known.

## 6. MSC-EVs Promote Dormancy of Tumor Cells

Some researchers have found that BMSC-EVs could decrease the proliferation of BM2 cells and reduce the abundance of stem cell-like surface markers. Further studies showed that dormant phenotypes were induced by overexpression of miR-23b in BM2 cells which suppressed MARCKS gene [51].

Another study has also indicated that stroma-derived exosomes contributed to breast cancer cells quiescence. The transfer of miRNAs might be involved in the dormancy of BM metastases [59]. Thus, targeting miRNA may be a valid therapeutic tool to reduce breast cancer metastasis.

## 7. MSC-EVs Promote Drug Resistance of Tumor Cells

It has been reported that BMSC-EVs not only increase MM cells growth but also induce resistance to bortezomib (BTZ), a proteasome inhibitor [57]. BMSC-EVs could inhibit the reduction of Bcl-2 expression caused by BTZ and reduce the cleavage of caspase-9, caspase-3, and PARP. Researchers also found BMSC-EVs could decrease the sensitivity of BM2 cells to docetaxel, a common chemotherapy agent [51].

In addition, the EVs derived from rat bone marrow-derived MSCs (rBMSC-EVs) can protect the rat pheochromocytoma PC12 cells against the excitotoxicity induced by glutamate. In this study it was also revealed that rBMSC-EVs reduced the expression of Bax and Bcl-2. Inhibition of PI3K/Akt pathway could partially abrogate the protective effects [60].

## 8. Conclusion

MSC-EVs could mimic the effects of mesenchymal stem cells in tumor therapies. Compared with cells, MSC-EVs are much smaller and have a lower possibility of immune rejection and formation of tumor. Therefore, MSC-EVs represent a promising alternative that could overcome the limitations of cell-therapy approaches. Besides being therapeutic agents, MSC-EVs have been advocated as “natural” drug delivery vehicles [76–78]. These lipid vesicles could be engineered to deliver therapeutic agents to target sites. For instance, it has been reported that the EVs secreted by SR4987 cells primed with paclitaxel (SR4987PTX) delivered active drugs and inhibited human pancreatic adenocarcinoma cells proliferation in a dose-dependent manner [79]. However, several questions have to be answered before clinical application of MSC-EVs. Firstly, it is very important to carefully evaluate the safety issues. For MSC-EVs have been reported to promote tumor growth, it is necessary to verify what kind of tumors may benefit from the treatment and to which extent MSC-EVs contribute to the beneficial effects. Secondly, researchers should thoroughly characterize the content of MSC-EVs and identify what molecules shuttled by MSC-EVs would function. Thirdly, the technologies for the isolation, detection, characterization, and engineering of MSC-EVs need to be standardized for their clinical application. Meanwhile, MSC-EVs dose, optimal timing of MSC-EVs administration, and schedule of administration also need to be developed for effective usage of MSC-EVs.

In conclusion, although MSC-EVs open up a promising opportunity to develop new “biotech drugs” in malignant diseases, further investigation is still required in some areas.

## Figures and Tables

**Table 1:** Protein contents of MSC-EVs.

Source of EVs	Protein	Function	Reference
Human bone marrow-derived MSCs	CD13, CD29, CD44, CD73, CD105, CD81, CD63, CD90, CD9	Surface antigen	[39–41]
Human bone marrow-derived MSCs	PDGFRB, EGFR, TGFBI, IGF2R	MSCs self-renewal	[39]
Human bone marrow-derived MSCs	CTNNBI, RAC1, RAC2, CHP, PRKCB, PPP2RIA, CAMK2D, PRKACA, CAMK2G	MSCs self-renewal and differentiation, Wnt signaling pathway	[39]
Human bone marrow-derived MSCs	PPP2RIA, MAPK1, USP9X, COL1A2, CD105, ENG	MSCs differentiation, TGF*β* signaling pathway	[39]
Human bone marrow-derived MSCs	FLNA, HSPAB, CACNA2D1, CHP, FLNC, PDGFRB, RAP1B, RRAS2, MAP4K4, EGFR, RRAS, GNG12, RAC1, HSPAIA, CDC42, RAC2, NRAS, MAPKl, CD81, FLNB, HSPBl, PRKCB, PRKACA, RAP1A, GNAI2, CAVI, PRDX2, PPP2RIA, SOD1, ITGA1, LPAR1	MSCs differentiation, MAPK signaling pathway	[39]
Human bone marrow-derived MSCs	ILK, FABP5, ACSL4	MSCs differentiation, PPAR signaling pathway	[39]
Human bone marrow-derived MSCs	ENG, USP9X	MSCs differentiation, BMP signaling pathway	[39]
Human adipose tissue-derived MSCs	Neprilysin	Degrade intracellular and extracellular *β*-amyloid peptide in neuroblastoma cell lines	[42]
Human bone marrow-derived MSCs	TIA, TIAR, HuR	T cell internal antigen	[43]
Human bone marrow-derived MSCs	Stau1, Stau2	Involved in the transport and stability of mRNA	[43]
Human bone marrow-derived MSCs	Ago2	Involved in the miRNA transport and processing	[43]
Human umbilical cord-derived MSCs	Wnt4	Enhance the proliferation and migration	[43]
Human umbilical cord-derived MSCs	Angiogenin, IL-6, bFGF, UPAR, VEGF, MCP-1, VEGF R2, IGF-I	Promote angiogenesis	[44, 45]

**Table 2:** mRNAs expressed in MSC-EVs.

Source of EVs	mRNA	Function	Reference
Human bone marrow-derived MSCs	IGF-1R	Enhance cell proliferation	[46]
Human bone marrow-derived MSCs	RAX2, OR11H12, OR2M3, DDN, GRIN3A, NIN, BMP15, IBSP, MAGED2, EPX, HK3, COL4A2, CEACAM5, SCNN1G, PKD2L2,	Involved in cell differentiation	[47]
Human bone marrow-derived MSCs	CLOCK, IRF6, RAX2, TCFP2, BCL6B	Involved in transcription	[47]
Human bone marrow-derived MSCs	HMGN4, TOPORS, ESF1, ELP4, POLR2E, HNRPH2	DNA/RNA binding	[47]
Human bone marrow-derived MSCs	SENP2, RBL1, CDC14B, S100A13	Cell cycle	[47]
Human bone marrow-derived MSCs	CEACAM5, CLEC2A, CXCR7	Receptors	[47]
Human bone marrow-derived MSCs	ADAM15, FUT3, ADM2, LTA4H, BDH2, RAB5A	Involved in metabolism	[47]
Human bone marrow-derived MSCs	CRLF1, IL1RN, MT1X	Immune regulation	[47]
Human bone marrow-derived MSCs	DDN, MSN, CTNNA1	Cytoskeleton	[47]
Human bone marrow-derived MSCs	COL4A2, IBSP	Extracellular matrix	[47]
Porcine adipose tissue-derived MSCs	FOXP3, JMJD1C, KDM6B	Encode transcription factors involved in chromosome organization	[48]
Porcine adipose tissue-derived MSCs	MDM4, IFT57, PEG3, PDCD4	Encode transcription factors involved in apoptosis	[48]
Porcine adipose tissue-derived MSCs	HGF, HES1, TCF4	Encode transcription factors involved in proangiogenic pathways	[48]
Porcine adipose tissue-derived MSCs	ZBTB1, ZNF217, ZNF238, ZNF461, ZNF568, ZNF667, ZHX1	Encode zinc-finger transcription factors	[48]
Porcine adipose tissue-derived MSCs	TMF1, BAZ2B, JMJD1C, MYNN, NFKBIZ, PEG3, KCNH6, RUNX1T1, SUFU	Encode transcription factors involved in alternative splicing	[48]

**Table 3:** miRNAs expressed in MSC-EVs.

Source of EVs	miRNA	Function	Reference
Human bone marrow-derived MSCs	miRNA-199b, miRNA-218, miRNA-148a, miRNA-135b, miRNA-221	Regulate osteoblast differentiation	[49]
Rats bone marrow-derived MSCs	miRNA-133b	Contribute to neurite outgrowth	[38]
Human bone marrow-derived MSCs	miRNA-15a	Inhibit the growth of multiple myeloma cells	[50]
Porcine adipose tissue-derived MSCs	miRNA-148a, miR532-5p, miRNA-378, let-7f	Regulate apoptosis, proteolysis angiogenesis, and cellular transport	[48]
Human bone marrow-derived MSCs	miRNA-21, miRNA-34a	Regulate cell survival and proliferation	[41]
Human bone marrow-derived MSCs	miRNA-23b	Induce dormant phenotypes	[51]
Mouse bone marrow-derived MSCs	miRNA-16	Target VEGF; suppress angiogenesis	[52]
Human adipose-derived MSCs	miRNA-486-5p, miRNA-10a-5p, let-7a-5p, miRNA-10b-5p, miRNA-191-5p, miRNA-22-3p, miRNA-222-3p, miRNA-21-5p, let-7f -5p, miRNA-127-3p, miRNA-143-3p, miRNA-99b-5p, miRNA-100-5p, miRNA-92a-3p, miRNA-92b-3p, miRNA-146a-5p, miRNA-26a-5p, miRNA-4485, miRNA-146b-5p, miRNA-51a-3p	Promote the migration; involved in replicative senescence, immune modulatory function; regulate cell cycle progression and proliferation; modulate angiogenesis	[53]
Human bone marrow-derived MSCs	miRNA-143-3p, miRNA-10b-5p, miRNA-486-5p, let-7a-5p, miRNA-22-3p, miRNA-21-5p, miRNA-222-3p, miRNA-28-3p, miRNA-191-5p, miRNA-100-5p, miRNA-99b-5p, miRNA-92a-3p, miRNA-127-3p, let-7f-5p, miRNA-92b-3p, miRNA-423-5p, let-7i-5p, miRNA-10a-5p, miRNA-27b-3p, miRNA-125b-5p	Promote the migration; involved in ASC replicative senescence, immune modulatory function; regulate cell cycle progression and proliferation; modulate angiogenesis	[53]

**Table 4:** Various effects of MSC-EVs on different types of tumor.

Source of EVs	Receptor cells	Biological function	Proposed mechanism	Reference
Human bone marrow-derived MSCs	Breast cancer cell line MCF7	Support breast tumor growth in vivo	Transport tumor supportive miRNA-21 and 34a	[41]
Human bone marrow-derived MSCs	HepG2 hepatoma, Kaposi's sarcoma, and Skov-3 ovarian tumor cell lines	Inhibit in vitro cell growth and survival of different tumor cell lines	Inhibit cell cycle progression in all cell lines and induce apoptosis in HepG2 and Kaposi's cells and necrosis in Skov-3	[54]
Human umbilical cord Wharton's jelly MSCs	Bladder tumor T24 cells	Inhibit T24 cells proliferative viability and induce apoptosis in T24 cells in vitro and in vivo	Downregulate phosphorylation of Akt protein kinase and upregulate cleaved caspase-3	[55]
Mouse bone marrow-derived MSCs	Mouse breast cancer cell line (4T1)	Suppress angiogenesis in vitro and in vivo	The exosome-derived miRNA-16 reduce the expression of VEGF in 4T1 cells	[52]
Human bone marrow-derived MSCs	Multiple myeloma cells	MM BMSC-EVs promote MM tumor growth; normal BMSC-EVs inhibit the growth of MM cells	The tumor suppressor miRNA-15a is present in normal BMSCs, but absent in MM BMSCs	[50]
Human bone marrow-derived MSCs	Human colon cancer cells, human gastric carcinoma cells, human lung fibroblast cell line	Promote tumor growth in vivo	Exosomes enhance VEGF expression in tumor cells by activating ERK1/2 pathway	[56]
Human bone marrow-derived MSCs, murine bone marrow-derived MSCs	Murine MM cells, human MM cells	Induce proliferation, migration, survival, and drug resistance of MM cells	Influence the activation of several survival relevant pathways, including c-Jun N-terminal kinase, p38, p53, and Akt	[57]
Human Wharton's Jelly MSCs	Human renal cancer cell	Promote the growth and aggressiveness of human renal cancer cell both in vitro and in vivo	Induce HGF synthesis via RNA transferred by EVs activating AKT and ERK1/2 signaling	[58]
Human bone marrow-derived MSCs	Human breast cancer cell line (BM2)	Promote breast cancer cells dormancy, drug resistance	Overexpression of miR-23b in BM2 cells induces dormant phenotypes through the suppression of a target gene, MARCKS	[51]
Human bone marrow-derived MSCs	Breast cancer cells MDA-MB-231 and T47D	Contribute to breast cancer cell quiescence	Transfer miRNAs from bone marrow stroma to breast cancer cells	[59]
Rat bone marrow-derived MSCs	Rat pheochromocytoma PC12 cells	Protect rat pheochromocytoma PC12 cells from glutamate-induced excitotoxicity	Upregulate Akt phosphorylation and Bcl-2 expression, downregulate Bax expression, and reduce the cleavage of caspase-3	[60]

## References

[1] Ji J. F., He B. P., Dheen S. T., Tay S. S. (2004). Interactions of chemokines and chemokine receptors mediate the migration of mesenchymal stem cells to the impaired site in the brain after hypoglossal nerve injury. *Stem Cells*.

[2] Hao N. B., Li C. Z., Lü M. H. (2015). SDF-1/CXCR4 axis promotes MSCs to repair liver injury partially through trans-differentiation and fusion with hepatocytes. *Stem Cells International*.

[3] Zhang L. X., Shen L. L., Ge S. H. (2015). Systemic BMSC homing in the regeneration of pulp-like tissue and the enhancing effect of stromal cell-derived factor-1 on BMSC homing. *International Journal of Clinical and Experimental Pathology*.

[4] Herrera M. B., Bussolati B., Bruno S. (2007). Exogenous mesenchymal stem cells localize to the kidney by means of CD44 following acute tubular injury. *Kidney International*.

[5] Tögel F., Hu Z., Weiss K., Isaac J., Lange C., Westenfelder C. (2005). Administered mesenchymal stem cells protect against ischemic acute renal failure through differentiation-independent mechanisms. *American Journal of Physiology—Renal Physiology*.

[6] Corcione A., Benvenuto F., Ferretti E. (2006). Human mesenchymal stem cells modulate B-cell functions. *Blood*.

[7] Beyth S., Borovsky Z., Mevorach D. (2005). Human mesenchymal stem cells alter antigen-presenting cell maturation and induce T-cell unresponsiveness. *Blood*.

[8] Jiang X. X., Zhang Y., Liu B. (2005). Human mesenchymal stem cells inhibit differentiation and function of monocyte-derived dendritic cells. *Blood*.

[9] Farini A., Sitzia C., Erratico S., Meregalli M., Torrente Y. (2014). Clinical applications of mesenchymal stem cells in chronic diseases. *Stem Cells International*.

[10] Khanabdali R., Saadat A., Fazilah M. (2015). Promoting effect of small molecules in cardiomyogenic and neurogenic differentiation of rat bone marrow-derived mesenchymal stem cells. *Drug Design, Development and Therapy*.

[11] Eirin A., Lerman L. O. (2014). Mesenchymal stem cell treatment for chronic renal failure. *Stem Cell Research and Therapy*.

[12] Van Poll D., Parekkadan B., Cho C. H. (2008). Mesenchymal stem cell–derived molecules directly modulate hepatocellular death and regeneration *in vitro* and *in vivo*. *Hepatology*.

[13] Wang F., Yasuhara T., Shingo T. (2010). Intravenous administration of mesenchymal stem cells exerts therapeutic effects on parkinsonian model of rats: focusing on neuroprotective effects of stromal cell-derived factor-1*α*. *BMC Neuroscience*.

[14] Tang J., Xie Q., Pan G., Wang J., Wang M. (2006). Mesenchymal stem cells participate in angiogenesis and improve heart function in rat model of myocardial ischemia with reperfusion. *European Journal of Cardio-Thoracic Surgery*.

[15] Prabakar K. R., Domínguez-Bendala J., Molano R. D. (2012). Generation of glucose-responsive, insulin-producing cells from human umbilical cord blood-derived mesenchymal stem cells. *Cell Transplantation*.

[16] Kidd S., Spaeth E., Dembinski J. L. (2009). Direct evidence of mesenchymal stem cell tropism for tumor and wounding microenvironments using in vivo bioluminescent imaging. *Stem Cells*.

[17] Deng J., Zou Z. M., Zhou T. L. (2011). Bone marrow mesenchymal stem cells can be mobilized into peripheral blood by G-CSF in vivo and integrate into traumatically injured cerebral tissue. *Neurological Sciences*.

[18] Kidd S., Spaeth E., Watson K. (2012). Origins of the tumor microenvironment: quantitative assessment of adipose-derived and bone marrow-derived stroma. *PLoS ONE*.

[19] Beckermann B. M., Kallifatidis G., Groth A. (2008). VEGF expression by mesenchymal stem cells contributes to angiogenesis in pancreatic carcinoma. *British Journal of Cancer*.

[20] Roorda B. D., ter Elst A., Kamps W. A., de Bont E. S. J. M. (2009). Bone marrow-derived cells and tumor growth: contribution of bone marrow-derived cells to tumor micro-environments with special focus on mesenchymal stem cells. *Critical Reviews in Oncology/Hematology*.

[21] Yu F. X., Hu W. J., He B., Zheng Y. H., Zhang Q. Y., Chen L. (2015). Bone marrow mesenchymal stem cells promote osteosarcoma cell proliferation and invasion. *World Journal of Surgical Oncology*.

[22] Swamydas M., Ricci K., Rego S. L., Dréau D. (2013). Mesenchymal stem cell-derived CCL-9 and CCL-5 promote mammary tumor cell invasion and the activation of matrix metalloproteinases. *Cell Adhesion and Migration*.

[23] Djouad F., Plence P., Bony C. (2003). Immunosuppressive effect of mesenchymal stem cells favors tumor growth in allogeneic animals. *Blood*.

[24] Zhu W., Xu W., Jiang R. (2006). Mesenchymal stem cells derived from bone marrow favor tumor cell growth in vivo. *Experimental and Molecular Pathology*.

[25] Secchiero P., Zorzet S., Tripodo C. (2010). Human bone marrow mesenchymal stem cells display anti-cancer activity in SCID mice bearing disseminated non-hodgkin's lymphoma xenografts. *PLoS ONE*.

[26] Qiao L., Xu Z., Zhao T. (2008). Suppression of tumorigenesis by human mesenchymal stem cells in a hepatoma model. *Cell Research*.

[27] Khakoo A. Y., Pati S., Anderson S. A. (2006). Human mesenchymal stem cells exert potent antitumorigenic effects in a model of Kaposi's sarcoma. *Journal of Experimental Medicine*.

[28] Ohlsson L. B., Varas L., Kjellman C., Edvardsen K., Lindvall M. (2003). Mesenchymal progenitor cell-mediated inhibition of tumor growth in vivo and in vitro in gelatin matrix. *Experimental and Molecular Pathology*.

[29] Li L., Tian H., Chen Z., Yue W., Li S., Li W. (2011). Inhibition of lung cancer cell proliferation mediated by human mesenchymal stem cells. *Acta Biochimica et Biophysica Sinica*.

[30] Rhee K. J., Lee J. I., Eom Y. W. (2015). Mesenchymal stem cell-mediated effects of tumor support or suppression. *International Journal of Molecular Sciences*.

[31] Nawaz M., Fatima F., Vallabhaneni K. C. (2016). Extracellular vesicles: evolving factors in stem cell biology. *Stem Cells International*.

[32] Rani S., Ryan A. E., Griffin M. D., Ritter T. (2015). Mesenchymal stem cell-derived extracellular vesicles: toward cell-free therapeutic applications. *Molecular Therapy*.

[33] Tan X., Gong Y. Z., Wu P., Liao D. F., Zheng X. L. (2014). Mesenchymal stem cell-derived microparticles: a promising therapeutic strategy. *International Journal of Molecular Sciences*.

[34] Bruno S., Camussi G. (2013). Role of mesenchymal stem cell-derived microvesicles in tissue repair. *Pediatric Nephrology*.

[35] Zhang Y., Chopp M., Meng Y. (2015). Effect of exosomes derived from multipluripotent mesenchymal stromal cells on functional recovery and neurovascular plasticity in rats after traumatic brain injury. *Journal of Neurosurgery*.

[36] Lindoso R. S., Collino F., Bruno S. (2014). Extracellular vesicles released from mesenchymal stromal cells modulate miRNA in renal tubular cells and inhibit ATP depletion injury. *Stem Cells and Development*.

[37] Xin H., Li Y., Buller B. (2012). Exosome-mediated transfer of miR-133b from multipotent mesenchymal stromal cells to neural cells contributes to neurite outgrowth. *Stem Cells*.

[38] Katsuda T., Ochiya T. (2015). Molecular signatures of mesenchymal stem cell-derived extracellular vesicle-mediated tissue repair. *Stem Cell Research and Therapy*.

[39] Kim H. S., Choi D. Y., Yun S. J. (2012). Proteomic analysis of microvesicles derived from human mesenchymal stem cells. *Journal of Proteome Research*.

[40] Ramos T. L., Sánchez-Abarca L. I., Muntión S. (2016). MSC surface markers (CD44, CD73, and CD90) can identify human MSC-derived extracellular vesicles by conventional flow cytometry. *Cell Communication and Signaling*.

[41] Vallabhaneni K. C., Penfornis P., Dhule S. (2015). Extracellular vesicles from bone marrow mesenchymal stem/stromal cells transport tumor regulatory microRNA, proteins, and metabolites. *Oncotarget*.

[42] Katsuda T., Tsuchiya R., Kosaka N. (2013). Human adipose tissue-derived mesenchymal stem cells secrete functional neprilysin-bound exosomes. *Scientific Reports*.

[43] Collino F., Deregibus M. C., Bruno S. (2010). Microvesicles derived from adult human bone marrow and tissue specific mesenchymal stem cells shuttle selected pattern of miRNAs. *PLoS ONE*.

[44] Zhang B., Wang M., Gong A. (2015). HucMSc-exosome mediated-Wnt4 signaling is required for cutaneous wound healing. *Stem Cells*.

[45] Chen J., Liu Z., Hong M. M. (2014). Proangiogenic compositions of microvesicles derived from human umbilical cord mesenchymal stem cells. *PLoS ONE*.

[46] Tomasoni S., Longaretti L., Rota C. (2013). Transfer of growth factor receptor mRNA via exosomes unravels the regenerative effect of mesenchymal stem cells. *Stem Cells and Development*.

[47] Bruno S., Grange C., Deregibus M. C. (2009). Mesenchymal stem cell-derived microvesicles protect against acute tubular injury. *Journal of the American Society of Nephrology*.

[48] Eirin A., Riester S. M., Zhu X. Y. (2014). MicroRNA and mRNA cargo of extracellular vesicles from porcine adipose tissue-derived mesenchymal stem cells. *Gene*.

[49] Xu J. F., Yang G. H., Pan X. H. (2014). Altered microRNA expression profile in exosomes during osteogenic differentiation of human bone marrow-derived mesenchymal stem cells. *PLoS ONE*.

[50] Roccaro A. M., Sacco A., Maiso P. (2013). BM mesenchymal stromal cell-derived exosomes facilitate multiple myeloma progression. *Journal of Clinical Investigation*.

[51] Ono M., Kosaka N., Tominaga N. (2014). Exosomes from bone marrow mesenchymal stem cells contain a microRNA that promotes dormancy in metastatic breast cancer cells. *Science Signaling*.

[52] Lee J. K., Park S. R., Jung B. K. (2013). Exosomes derived from mesenchymal stem cells suppress angiogenesis by down-regulating VEGF expression in breast cancer cells. *PLoS ONE*.

[53] Baglio S. R., Rooijers K., Koppers-Lalic D. (2015). Human bone marrow- and adipose-mesenchymal stem cells secrete exosomes enriched in distinctive miRNA and tRNA species. *Stem Cell Research and Therapy*.

[54] Bruno S., Collino F., Deregibus M. C., Grange C., Tetta C., Camussi G. (2013). Microvesicles derived from human bone marrow mesenchymal stem cells inhibit tumor growth. *Stem Cells and Development*.

[55] Wu S., Ju G. Q., Du T., Zhu Y. J., Liu G.-H. (2013). Microvesicles derived from human umbilical cord Wharton's jelly mesenchymal stem cells attenuate bladder tumor cell growth in vitro and in vivo. *PLoS ONE*.

[56] Zhu W., Huang L., Li Y. (2012). Exosomes derived from human bone marrow mesenchymal stem cells promote tumor growth in vivo. *Cancer Letters*.

[57] Wang J., Hendrix A., Hernot S. (2014). Bone marrow stromal cell-derived exosomes as communicators in drug resistance in multiple myeloma cells. *Blood*.

[58] Du T., Ju G., Wu S. (2014). Microvesicles derived from human Wharton's jelly mesenchymal stem cells promote human renal cancer cell growth and aggressiveness through induction of hepatocyte growth factor. *PLoS ONE*.

[59] Lim P. K., Bliss S. A., Patel S. A. (2011). Gap junction-mediated import of microRNA from bone marrow stromal cells can elicit cell cycle quiescence in breast cancer cells. *Cancer Research*.

[60] Lin S. S., Zhu B., Guo Z. K. (2014). Bone marrow mesenchymal stem cell-derived microvesicles protect rat pheochromocytoma PC12 cells from glutamate-induced injury via a PI3K/Akt dependent pathway. *Neurochemical Research*.

[61] Keller S., Sanderson M. P., Stoeck A., Altevogt P. (2006). Exosomes: from biogenesis and secretion to biological function. *Immunology Letters*.

[62] Ratajczak J., Wysoczynski M., Hayek F., Janowska-Wieczorek A., Ratajczak M. Z. (2006). Membrane-derived microvesicles: important and underappreciated mediators of cell-to-cell communication. *Leukemia*.

[63] Tan S. S., Yin Y., Lee T. (2017). Therapeutic MSC exosomes are derived from lipid raft microdomains in the plasma membrane. *Journal of Extracellular Vesicles*.

[64] Yáñez-Mó M., Siljander P. R., Andreu Z. (2015). Biological properties of extracellular vesicles and their physiological functions. *Journal of Extracellular Vesicles*.

[65] Lopatina T., Gai C., Deregibus M. C., Kholia S., Camussi G. (2016). Cross talk between cancer and mesenchymal stem cells through extracellular vesicles carrying nucleic acids. *Frontiers in Oncology*.

[66] Biancone L., Bruno S., Deregibus M. C., Tetta C., Camussi G. (2012). Therapeutic potential of mesenchymal stem cell-derived microvesicles. *Nephrology Dialysis Transplantation*.

[67] Lai R. C., Tan S. S., Teh B. J. (2012). Proteolytic potential of the MSC exosome proteome: implications for an exosome-mediated delivery of therapeutic proteasome. *International Journal of Proteomics*.

[68] Bartel D. P. (2004). MicroRNAs: genomics, biogenesis, mechanism, and function. *Cell*.

[69] Zhang J., Li S., Li L. (2015). Exosome and exosomal microRNA: trafficking, sorting, and function. *Genomics, Proteomics and Bioinformatics*.

[70] Li J., Dong J., Zhang Z. H. (2013). miR-10a restores human mesenchymal stem cell differentiation by repressing KLF4. *Journal of Cellular Physiology*.

[71] Chen T. S., Lai R. C., Lee M. M., Choo A. B. H., Lee C. N., Lim S. K. (2009). Mesenchymal stem cell secretes microparticles enriched in pre-microRNAs. *Nucleic Acids Research*.

[72] Nagpal N., Kulshreshtha R. (2014). miR-191: an emerging player in disease biology. *Frontiers in Genetics*.

[73] Xin H., Li Y., Liu Z. (2013). MiR-133b promotes neural plasticity and functional recovery after treatment of stroke with multipotent mesenchymal stromal cells in rats via transfer of exosome-enriched extracellular particles. *Stem Cells*.

[74] Feng Y., Huang W., Wani M., Yu X., Ashraf M. (2014). Ischemic preconditioning potentiates the protective effect of stem cells through secretion of exosomes by targeting Mecp2 via miR-22. *PLoS ONE*.

[75] Katakowski M., Buller B., Zheng X. (2013). Exosomes from marrow stromal cells expressing miR-146b inhibit glioma growth. *Cancer Letters*.

[76] Ohno S. I., Drummen G. P. C., Kuroda M. (2016). Focus on extracellular vesicles: development of extracellular vesicle-based therapeutic systems. *International Journal of Molecular Sciences*.

[77] Han C., Sun X., Liu L. (2016). Exosomes and their therapeutic potentials of stem cells. *Stem Cells International*.

[78] Katsuda T., Kosaka N., Takeshita F., Ochiya T. (2013). The therapeutic potential of mesenchymal stem cell-derived extracellular vesicles. *Proteomics*.

[79] Pascucci L., Coccè V., Bonomi A. (2014). Paclitaxel is incorporated by mesenchymal stromal cells and released in exosomes that inhibit in vitro tumor growth: a new approach for drug delivery. *Journal of Controlled Release*.

